# Mental fatigue and return to well-being in athletes: roles of cognitive reappraisal, gender, and alcohol consumption

**DOI:** 10.3389/fpsyg.2025.1569973

**Published:** 2025-04-15

**Authors:** Mehmet Kara, Emre Bulent Ogras, Laurentiu-Gabriel Talaghir, Teodora Mihaela Iconomescu, Huseyin Gumus

**Affiliations:** ^1^Faculty of Sports Sciences, Mersin University, Mersin, Turkey; ^2^Faculty of Physical Education and Sport, Dunarea de Jos University of Galati, Galati, Romania

**Keywords:** mental fatigue, cognitive reappraisal, recovery, gender, alcohol use

## Abstract

This study aimed to investigate the impact of mental fatigue awareness on psychological recovery processes in athletes. It also examines the mediating role of cognitive reappraisal and the moderating effects of gender and alcohol use. A sample of 639 active athletes from diverse regions of Turkey participated in the online study. Data were collected through the Sport Psychological Recovery Scale, the Emotion Regulation Scale for Athletes, and the Mental Fatigue Awareness Scale in Athletes. The findings indicate that mental fatigue awareness has a direct, negative effect on recovery (β = −0.2526, *p* < 0.001). Also, cognitive reappraisal partially mediates this relationship (β = −0.236, *p* < 0.001). Gender was identified as a significant moderator, with women exhibiting a stronger negative impact of mental fatigue awareness on their recovery processes compared to men. However, the moderating effect of alcohol consumption was not statistically significant. The study contributes to the understanding of the interplay between mental fatigue awareness and recovery in athletes, highlighting the influence of cognitive and emotional regulation and the importance of gender as a moderating variable. Future research should employ longitudinal designs and larger samples to explore these relationships in greater depth.

## 1 Introduction

Athletes undergo rigorous training regimes to achieve optimal performance, necessitating the development of both physical and mental resilience. High-level athletic performance depends not only on physical strength and technical proficiency but also on the effective management of cognitive and emotional resources. Mental preparation, often regarded as a cognitively demanding process, can be compromised by intensive physical training. Specifically, excessive physical exertion may impair cognitive abilities, leading to reduced concentration, diminished motivation, increased distractibility, and forgetfulness, all of which are characteristic of mental fatigue (Kara et al., 2024).

Mental fatigue is marked by the weakening of cognitive processes, including attention, focus, and motivation, leading to a decline in cognitive performance. In sports, mental fatigue can result from prolonged and intense training or frequent competition. Existing literature underscores the detrimental effects of mental fatigue on decision-making, reaction time, and general cognitive function, all of which are critical in competitive sports (Grillon et al., 2015; Tanaka, 2015; Pires et al., 2018; Gecaite-Stonciene et al., 2020; Chen et al., 2024). It's important to distinguish mental fatigue from physical fatigue as it can develop independently and exert unique effects. For instance, Marcora et al. (2009) demonstrated that mental fatigue increases perceived effort during physical tasks, decreasing performance, without causing physiological changes. In a similar vein, Holgado et al. (2023) found that mental fatigue can reduce neuromuscular function in athletes, thus affecting exercise performance. Mental fatigue is also associated with alterations in brain regions responsible for attention and cognitive control (Ishii et al., 2014).

Beyond its impact on physical capacities, mental fatigue influences key cognitive processes necessary for athletic success, including decision-making and attentional control. Effective decision-making in sports involves evaluating multiple options and selecting the most optimal course of action (Çetin and Kara, 2024). However, mentally fatigued athletes may experience difficulties in this process due to impaired attentional resources and an increased perception of cognitive effort. Emotion regulation strategies, particularly cognitive reappraisal and emotional suppression, are crucial in helping athletes regulate their emotional states effectively, ultimately shaping their performance outcomes (Tamir, 2016; Matsumoto et al., 2008). Awareness of mental fatigue may thus enhance an athlete's ability to regulate emotions, thereby supporting psychological recovery.

Psychological recovery refers to an athlete's ability to rebound from stressors, adversity, or negative experiences through adaptive cognitive, emotional, and behavioral adjustments (Loch et al., 2020). Being aware of and dealing with mental fatigue may help the recovery process by encouraging the use of adaptive emotion regulation strategies like cognitive reappraisal. Additionally, mindfulness-based practices have been suggested as a means to enhance emotional regulation, resilience, and overall performance in athletes. While the effects of mental fatigue and psychological recovery have been widely studied, limited research has explored how demographic and personal characteristics, particularly gender and alcohol consumption, influence these processes. The inclusion of these variables is theoretically grounded in well-established psychological frameworks.

The Gender Schema Theory (Bem, 1981) has been widely studied regarding how gender influences cognition and emotional processing. This theory suggests that individuals develop mental models that shape their perceptions, behaviors, and responses to stressors. These schemas influence how men and women experience and regulate emotions, which may subsequently impact their psychological recovery following mental fatigue. Research shows that women use cognitive reappraisal more often than men. On the other hand, men are more likely to use suppression strategies, which may make it harder for them to recover from stress that causes fatigue (Gross and John, 2003; Nolen-Hoeksema, 2012). Given that cognitive reappraisal is associated with enhanced psychological resilience, gender differences may play a moderating role in the relationship between mental fatigue awareness and recovery. Furthermore, neurocognitive studies indicate that gender differences in emotional processing may influence how athletes manage and recover from mental fatigue. Research by Wagstaff (2014) suggests that female athletes, on average, exhibit greater emotional awareness and self-regulation skills, which could facilitate faster psychological recovery from fatigue. In contrast, male athletes may experience greater difficulty disengaging from fatigue-induced cognitive stressors, thereby prolonging the recovery process. These findings underscore the need to examine gender as a moderating factor in the relationship between mental fatigue and psychological recovery.

The inclusion of alcohol consumption as a moderating variable is theoretically supported by research on its effects on executive functioning and self-regulation. Field et al. (2010) highlight that alcohol impairs cognitive control mechanisms, leading to reduced attentional capacity and decreased emotion regulation efficiency. Since self-regulation is a crucial factor in recovery from mental fatigue, alcohol consumption may moderate the extent to which athletes can engage in adaptive recovery strategies. Furthermore, alcohol consumption has been shown to negatively impact prefrontal cortex function, which governs self-regulation, decision-making, and cognitive flexibility (Sayette, 2017). This impairment may contribute to a reduced capacity for cognitive reappraisal, making it more challenging for athletes to recover from fatigue-related stress. Additionally, Woolsey et al. (2010) suggest that alcohol consumption is associated with increased risk-taking behavior in athletes, which may further exacerbate the negative consequences of mental fatigue. Given that psychological recovery relies on an athlete's ability to effectively regulate emotions and cognitive effort, individuals with higher alcohol consumption levels may exhibit delayed recovery due to compromised cognitive control. The type of sport also plays an important role in alcohol consumption patterns. Research suggests that team sports tend to be associated with higher alcohol use compared to individual sports (Fraczek et al., 2020). This suggests that the social environments created within team sports may normalize and even encourage drinking, contributing to greater alcohol consumption among team-sport athletes. The nature of sports participation significantly influences drinking habits, as some sports foster environments where alcohol use is more socially accepted (Halldorsson et al., 2014; Villalba et al., 2016).

Building on these theoretical foundations, this study investigates the role of cognitive reappraisal as a mediating factor between mental fatigue awareness and psychological recovery. Additionally, it examines the moderating effects of gender and alcohol consumption in this relationship. By addressing these unexplored relationships, the study aims to provide novel insights into the psychological mechanisms underlying mental fatigue and resilience in athletes. Understanding these dynamics is essential for developing targeted interventions that enhance both mental and physical performance in sports. Accordingly, the main aim of this study is to better understand the relationship between athletes' mental fatigue awareness and psychological recovery processes and to reveal the mediating role of cognitive reappraisal in this process. This goal was determined by considering the gaps in the existing literature and the needs related to athletes' performance sustainability.

## 2 Material and methods

This section details the study's methodology, including the participant pool, data collection tools, the research procedure, and statistical analyses. Ethical approval for the study was granted by the Mersin University Sports Sciences Ethics Committee (02/12/2024, number 073), adhering to the principles outlined in the 2008 Helsinki Declaration Ethical Standards.

### 2.1 Research model and study group

The study employed a predictive correlational model with data collected via convenience sampling, a non-probability sampling method. Individual and team athletes actively engaged in sports in Turkey as of December 2024 were recruited for the study. Non-probability sampling methods, such as convenience and purposive sampling, are typically employed when probability sampling is either impractical or costly (Dewi et al., 2019; Hannie et al., 2020; Robbins et al., 2021). A total of 639 athletes participated in the study, with 323 (50.54%) females and 316 (49.46%) males. Data was collected via an online Google Form and then transferred to statistical software packages for analysis.

### 2.2 Data collection tools

Three established scales were employed in this study.

#### 2.2.1 Sport psychological recovery scale (SPRS)

Developed by Kaygusuz and Karagözoglu (2023) specifically for athletes, this scale comprises 20 items across four factors measured on a 10-point scale. The factors include Mental Recovery, Vitality and Energy, Psychological Detachment, and Return to Well-being. The study only utilized the Return to Well-being subdimension for the mediation analysis.

#### 2.2.2 Emotion regulation scale for athletes (ERSA)

Adapted for Turkish culture by Tingaz and Ekiz (2021) from the original work by Gross and John (2003), this scale examines emotion regulation in athletes. The scale features 8 items across two subscales (Cognitive Reappraisal and Suppression), rated on a 7-point scale. The study utilized only the Cognitive Reappraisal subdimension, considered more pertinent to the model.

#### 2.2.3 Mental fatigue awareness scale in athletes (MFASA)

Developed by Kara et al. (2024), this single-dimension scale includes 25 items assessing mental fatigue awareness on a 5-point Likert scale. The scale demonstrated high internal consistency (Cronbach's alpha = 0.96).

### 2.3 Data analysis techniques

Research data were collected online from 677 active athletes via the above-listed tools. The gathered data were initially subjected to missing data analysis, outlier analysis, and assumption checks. As the observations were collected online, no missing data were detected. Data cleaning and assumption analyses led to the exclusion of 38 observations, resulting in a final sample of 639.

Initially, the 677 observations were transferred to statistical software packages, and the mode, median, and arithmetic mean were examined. Since these values were very close to each other, the distribution was determined to be normal. To test for outliers in the observation set, Mahalanobis distances and Z-score values were examined. All *Z*-scores were within the range of 2.86 to −3.47. Secondly, when Mahalanobis distances were evaluated based on the Chi-square distribution criterion (χ236; 0.001 = 73.402), 15 observations exceeding the table value were excluded from the analysis, reducing the initial 677 observations to 662. Based on the Tabachnick's criteria (−4, +4), we decided that there were no single outliers in the dataset. Subsequently, the dataset with 662 observations underwent outlier analysis via box plots and scatterplots. In this context, 23 observations were excluded, and the analysis proceeded with the remaining 639 observations.

#### 2.3.1 Normality and autocorrelation

To assess the normality of the final dataset, skewness and kurtosis values were examined. For the Mental Fatigue Awareness scale, the values were: skewness = 0.298, kurtosis = −0.354. For the ERSA Cognitive Reappraisal subscale: skewness = −0.218, kurtosis = −0.361. For the SPRS Return to Well-being subscale: skewness = −0.115, kurtosis = −0.595. Since these values fell within the ± 1.5 range, it was concluded that the dataset exhibited a normal distribution (George and Mallery, 2016). As for detecting autocorrelation of errors, the Durbin-Watson (D-W) value, including all items, was found to be 1.564, indicating that the errors were independent of each other (Kalayci, 2010).

#### 2.3.2 Multicollinearity

In order to detect multicollinearity, the variance inflation factor (VIF) was examined. The VIF values were found to be between 1.815 and 3.844. Tolerance values were also examined and found to be between 0.510 and 0.265. As all Tolerance values were above 0.20 and all VIF values were below 5, it was confirmed that there was no multicollinearity problem among the variables in the model (Raheem et al., 2019; Makrakis et al., 2024). Condition Index (CI) values were found to be 5.336 and 10.994. The observations in the dataset were transferred to Jamovi 2.6.2 software for additional analysis.

### 2.4 Research hypotheses

**Figure 1:**
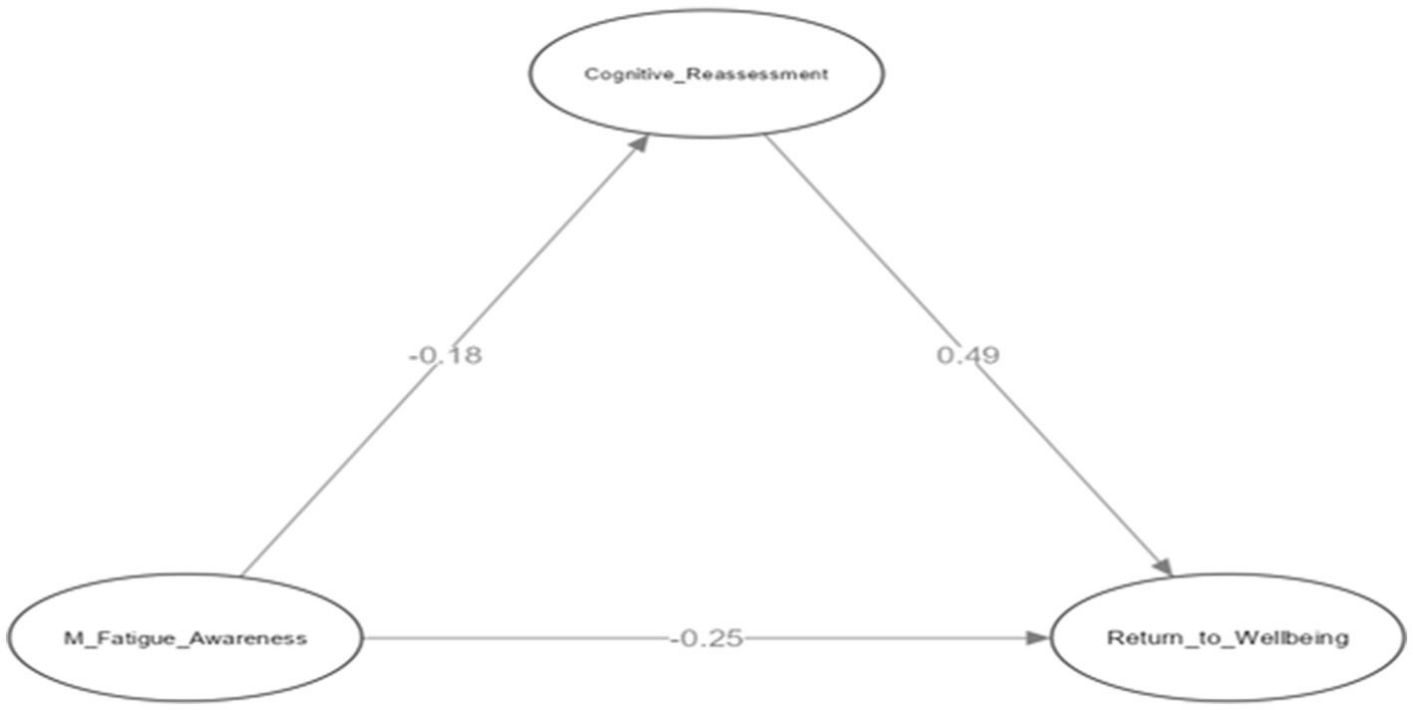
Research model diagram.

We investigated a mediation model with Mental Fatigue Awareness as the independent variable, the Return to Well-Being subdimension of the SPRS as the dependent variable, and Cognitive Reappraisal of the ERSA as the mediating variable (Figure 1).

This model proposes that mental fatigue awareness can lead athletes to reduced focus on daily activities (Kara et al., 2024). Awareness of mental fatigue is posited as a trigger for recovery behavior. Psychological recovery, specifically return to well-being, encompasses psychological skills that enable athletes to withstand stress and enhance performance (Braun-Trocchio et al., 2022). This leads to the examination of cognitive reappraisal within the emotion regulation process, an essential factor in the relationship between mental fatigue and psychological recovery. Cognitive reappraisal reduces negative emotions by encouraging athletes to reinterpret stressful events more positively (Kobayashi et al., 2020; Han et al., 2023), and could allow a more adaptable perspective toward challenging situations and healthier coping strategies. Based on this framework, the following hypotheses were formulated:

**H**^**1**^: Mental fatigue awareness in athletes has a statistically significant negative effect on their return to well-being.

**H**^**2**^: Athletes' mental fatigue awareness has a statistically significant negative effect on their cognitive reappraisal.

**H**^**3**^: Athletes' cognitive reappraisal has a statistically significant positive effect on their return to well-being.

**H**^**4**^: Emotion regulation mediates the relationship between athletes' mental fatigue awareness and their psychological recovery.

The following additional hypotheses address potential moderating effects:

**H**^**5**^: Gender moderates the mediating effect of emotion regulation on the relationship between athletes' mental fatigue and their psychological recovery.

**H**^**6**^: Alcohol consumption status moderates the mediating effect of emotion regulation on the relationship between athletes' mental fatigue and their psychological recovery.

To identify situations where the relationship between the independent and dependent variables differs, this study conducted a moderation analysis to understand if a third variable (the moderator) influences the relationship between the main variables of interest. This involved analyzing the independent variable, the moderator variable, and the interaction term (created by multiplying the independent and moderator variables). The statistical significance of the interaction term was assessed to determine the presence and nature of the moderation effect.

## 3 Results

Table 1 presents the demographic characteristics of the 639 active athletes included in the study. Slightly more than half of the participants were female (50.54%). In terms of sports experience, 44.1% had been active in their sport for 1–5 years, 31.9% for 6–10 years, and 23.9% for 11 years or more. The majority of the athletes were young adults, with 75.43% aged 19–25 years old. Participants aged under 18 constituted 12.83% of the sample, while 11.74% were aged between 26 and 45.

**Table 1:** Descriptive statistics of demographic variables.

**Age**				**Duration of sports experience**			
				**1–5 years**	**6–10 years**	**11 and above**	**Total**
Under 18	Gender	Female	% Gender	21 (47.7%)	22 (50.0%)	1 (2.3%)	44 (100.0%)
		Male	% Gender	13 (34.2%)	18 (47.4%)	7 (18.4%)	38 (100.0%)
	Total			34 (41.5%)	40 (48.8%)	8 (9.8%)	82 (100.0%)
19–25 years	Gender	Female	% Gender	147 (58.1%)	56 (22.1%)	50 (19.8%)	253 (100.0%)
		Male	% Gender	84 (36.7%)	95 (41.5%)	50 (21.8%)	229 (100.0%)
	Total			231 (47.9%)	151 (31.3%)	100 (20.7%)	482 (100.0%)
26–45 years	Gender	Female	% Gender	6 (23.1%)	8 (30.8%)	12 (46.2%)	26 (100.0%)
		Male	% Gender	11 (22.4%)	5 (10.2%)	33 (67.3%)	49 (100.0%)
	Total			17 (22.7%)	13 (17.3%)	45 (60.0%)	75 (100.0%)
Total	Gender	Female	% Gender	174 (53.9%)	86 (26.6%)	63 (19.5%)	323 (100.0%)
		Male	% Gender	108 (34.2%)	118 (37.3%)	90 (28.5%)	316 (100.0%)
	Total			282 (44.1%)	204 (31.9%)	153 (23.9%)	639 (100.0%)

Table 2 presents the results derived from the confirmatory factor analysis (CFA) conducted to assess the validity of the measurement model. The results for each scale were as follows: The Mental Fatigue Awareness Scale demonstrated acceptable fit indices (χ^2^/d*f* = 7.29, CFI = 0.84, TLI = 0.83, RMSEA = 0.099, SRM*R* = 0.052) and excellent internal consistency (Cronbach's α = 0.96). The Cognitive Reappraisal dimension of the Emotion Regulation Scale exhibited excellent fit (χ^2^/d*f* = 0.66, CFI = 1.0, TLI = 1.0, RMSEA = 0.000, SRMR = 0.005) and good internal consistency (Cronbach's α = 0.86). Similarly, the Return to Well-being dimension of the Psychological Recovery Scale showed good fit (χ^2^/d*f* = 3.82, CFI = 0.98, TLI = 0.96, RMSEA = 0.066, SRMR = 0.020) and excellent internal consistency (Cronbach's α = 0.93).

**Table 2:** Results of confirmatory factor analysis.

	**CMIN/DF (*x*^2^/d*f*)**	**CFI**	**TLI**	**RMSEA**	**SRMR**	**Cronbach's alpha**
Mental fatigue awareness scale	2006/275 = 7.29	0.84	0.83	0.099	0.052	0.96
Emotion regulation scale for athletes (*cognitive reappraisal*)	1.32/2 = 0.66	1.0	1.0	0.000	0.005	0.86
Sport psychological recovery scale (*return to well-being*)	53.5/14 = 3.82	0.98	0.96	0.066	0.020	0.93

Correlation analysis was conducted to examine the relationships between the study variables in Table 3. Mental Fatigue Awareness was negatively associated with both Cognitive Reappraisal (*r* = −0.178) and Return to Well-being (*r* = −0.340), indicating that individuals with higher mental fatigue awareness tended to report lower levels of cognitive reappraisal and return to well-being. A moderate positive correlation was found between Cognitive Reappraisal and Return to Well-being (*r* = 0.536), suggesting that those who utilize cognitive reappraisal strategies are more likely to experience a greater sense of well-being.

**Table 3:** Correlation analysis results for factors and scales.

***N* = 639**	***X***	**SD**	**1**	**2**	**3**
1. Mental fatigue awareness	2.3812	0.780	1	−0.178^**^	−0.340^**^
2. Cognitive reappraisal	4.7480	1.34		1	0.536^**^
3. Return to well-being	6.3783	2.10			1

** *p* < *0.01*.

### 3.1 Findings on mediation model

You may insert up to 5 heading levels into your manuscript as can be seen in “Styles” tab of this template. These formatting styles are meant as a guide, as long as the heading levels are clear, Frontiers style will be applied during typesetting.

**Figure 2:**
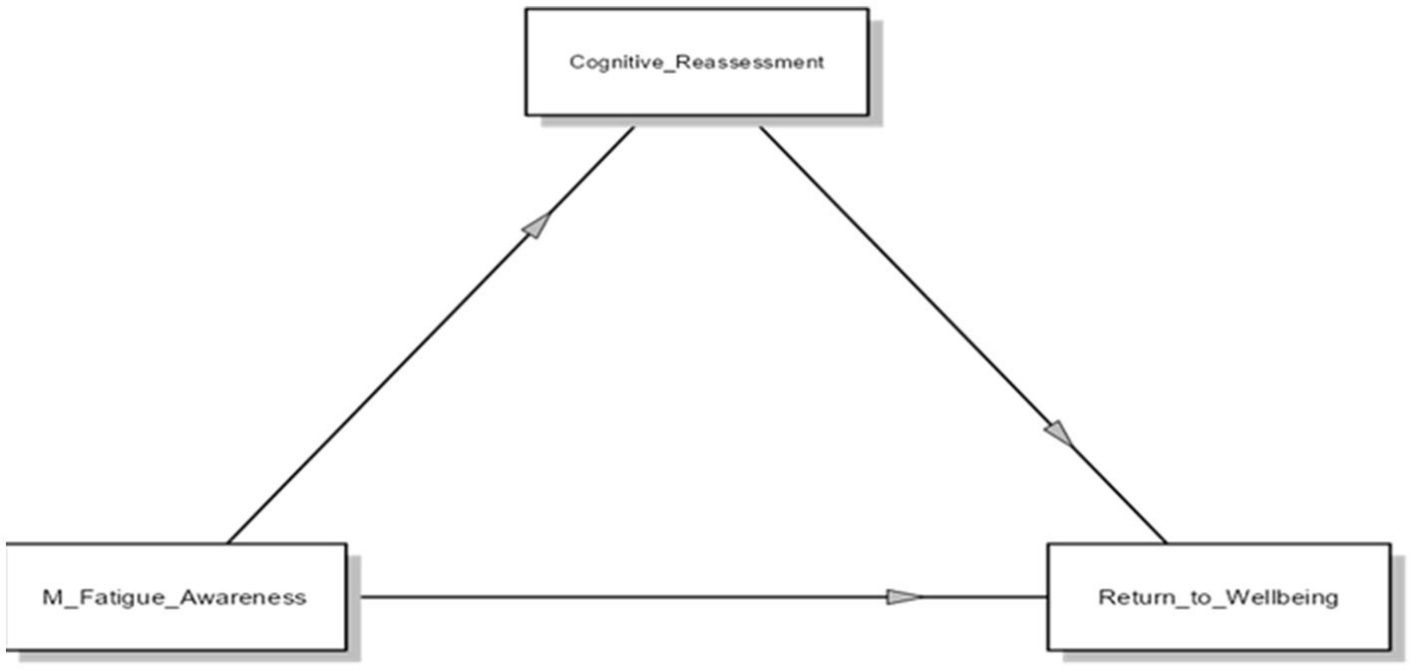
Research model diagram: beta coefficients.

Within the mediation analysis of this study, Mental Fatigue Awareness served as the independent variable, Return to Well-being as the dependent variable, and Cognitive Reappraisal as the mediator. The beta coefficients for this model are presented in Figure 2.

In mediation research, the concepts of direct and indirect effects are crucial for understanding the mechanisms by which an independent variable influences a dependent variable through a mediator. Direct effects describe the influence of the independent variable on the dependent variable that is not transmitted through the mediator (Daniel et al., 2015), while indirect effects represent the portion of the effect in the model that operates through the mediator (Preacher and Hayes, 2008). The findings of the mediation model are presented in Table 4.

**Table 4:** Mediation analysis of the relationship between mental fatigue awareness, cognitive reappraisal, and return to well-being.

	**Estimate**	**SH**	**β**	***Z***	***p***	**Mediation effect (%)**
Mental fatigue awareness → cognitive reappraisal	−0.306	0.0669	−0.0875	−4.58	<0.001	
Cognitive reappraisal → return to well-being	0.771	0.0509	0.4909	15.13	<0.001	
Mental fatigue awareness → return to well-being	−0.682	0.0875	−0.2526	−7.79	<0.001	
Indirect effect	−0.236	0.0539	−0.0875	−4.38	<0.001	25.7
Direct effect	−0.682	0.0875	−0.2526	−7.79	<0.001	74.3
Total effect	−0.917	0.1004	−0.3401	−9.14	<0.001	100.0

The study investigated the relationships between Mental Fatigue Awareness, Cognitive Reappraisal, and Return to Well-being. Table 4 lists the findings showing direct, indirect, and total effects among these variables.

#### 3.1.1 Direct effects

Mental Fatigue Awareness had a significant negative direct effect on Return to Well-being (β = −0.2526, *p* < 0.001), supporting Hypothesis 1 (H1). This suggests that increased awareness of mental fatigue negatively impacts individuals' recovery processes.

Mental Fatigue Awareness also had a significant negative direct effect on Cognitive Reappraisal (β = −0.0875, *p* < 0.001), confirming Hypothesis 2 (H2). This indicates that higher mental fatigue awareness leads to decreased levels of cognitive reappraisal.

Cognitive Reappraisal had a significant positive direct effect on Return to Well-being (β = 0.4909, *p* < 0.001), supporting Hypothesis 3 (H3). This highlights the positive role of effective cognitive reappraisal strategies in facilitating recovery.

#### 3.1.2 Indirect and total effects

Mental Fatigue Awareness had a significant indirect effect on Return to Well-being through Cognitive Reappraisal (β = −0.236, *p* < 0.001). This indirect effect accounted for approximately 25.7% of the total effect. However, the presence of a significant direct effect indicates partial mediation (Hayes, 2015).

The total effect of Mental Fatigue Awareness on Return to Well-being was β = −0.3401. Of this total effect, 74.3% was attributed to the direct effect, and 25.7% to the indirect pathway. Such findings demonstrate that mental fatigue awareness influences individuals' recovery processes both directly and indirectly through cognitive reappraisal. Cognitive reappraisal appears to play a crucial role in mitigating the negative effects of mental fatigue awareness.

**Figure 3:**
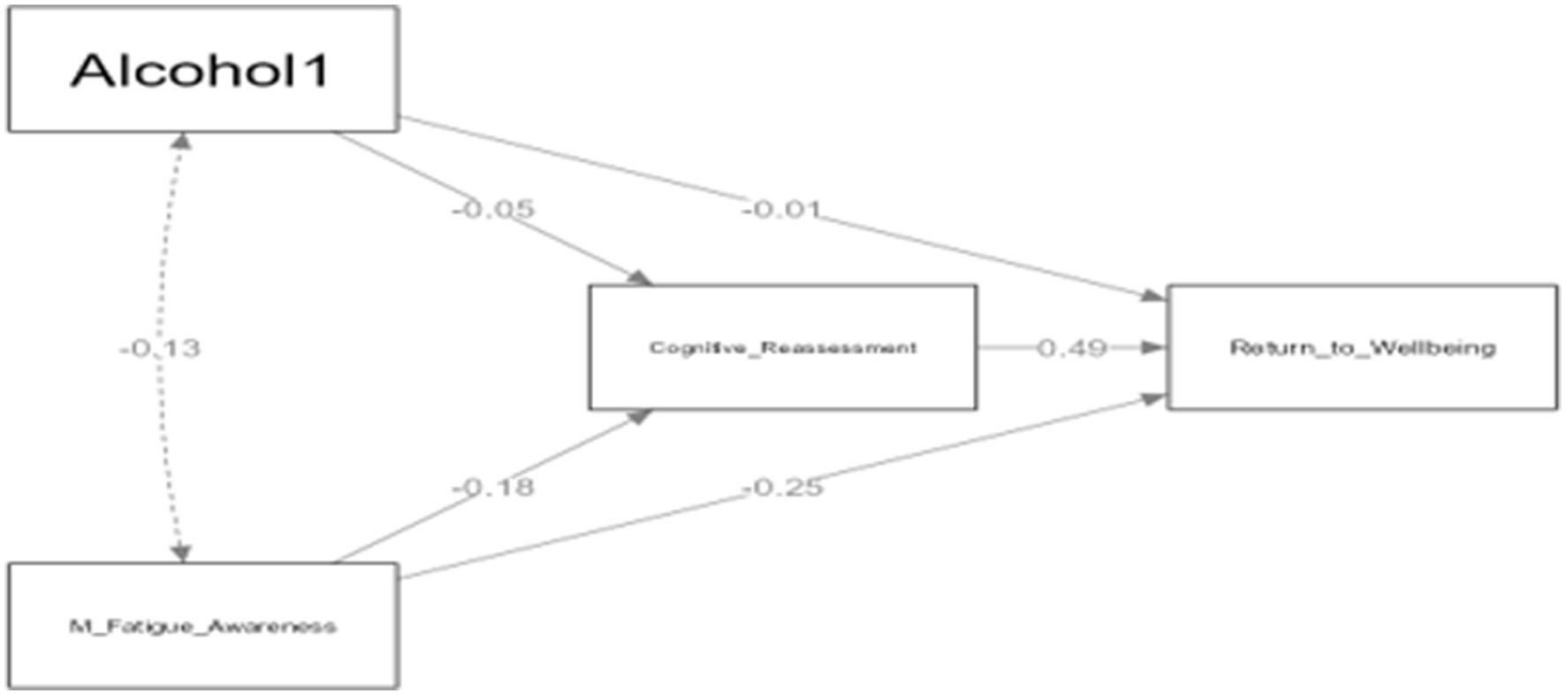
Situational effects of mental fatigue awareness on return to well-being by gender.

Table 5 presents an analysis of the relationship between mental fatigue awareness and return to well-being, specifically examining potential gender differences. For women, increased mental fatigue awareness was significantly associated with a less successful return to well-being (*b* = −1.0735, *p* < 0.01, 95% CI = [−1.34, −0.79]). A similar negative association was observed for men (*b* = −0.7230, *p* < 0.01, 95% CI = [−1.01, −0.43]). However, the magnitude of this effect was greater for women, indicating a heightened sensitivity to the impact of mental fatigue awareness on their return to well-being. Bootstrap confidence intervals confirmed the reliability of these findings, as neither interval included zero. These results suggest that gender plays a moderating role in the relationship between mental fatigue awareness and return to well-being. Women appear to be more vulnerable to the negative consequences of heightened mental fatigue awareness on their recovery process. Figure 3 provides a visual representation of these gender-specific effects.

**Table 5:** Standardized coefficients and 95% bootstrap confidence intervals.

**Gender**	**Estimation (*b*)**	**SE**	***t***	**LB**	**UB**	***p***
Female	−1.0735^**^	0.1407	−7.63^**^	1.34	−0.79	0.000
Male	−0.7230^**^	0.1472	−4.91^**^	1.01	−0.43	0.000

* *p* < *0.05*,

** *p* < *0.01*.

Figure 3 shows that the effect of mental fatigue awareness on return to well-being is more pronounced for women (1), and this trend shows a less steep decline for men (2). This visualization clearly demonstrates the differentiation of effects depending on gender.

**Figure 4:**
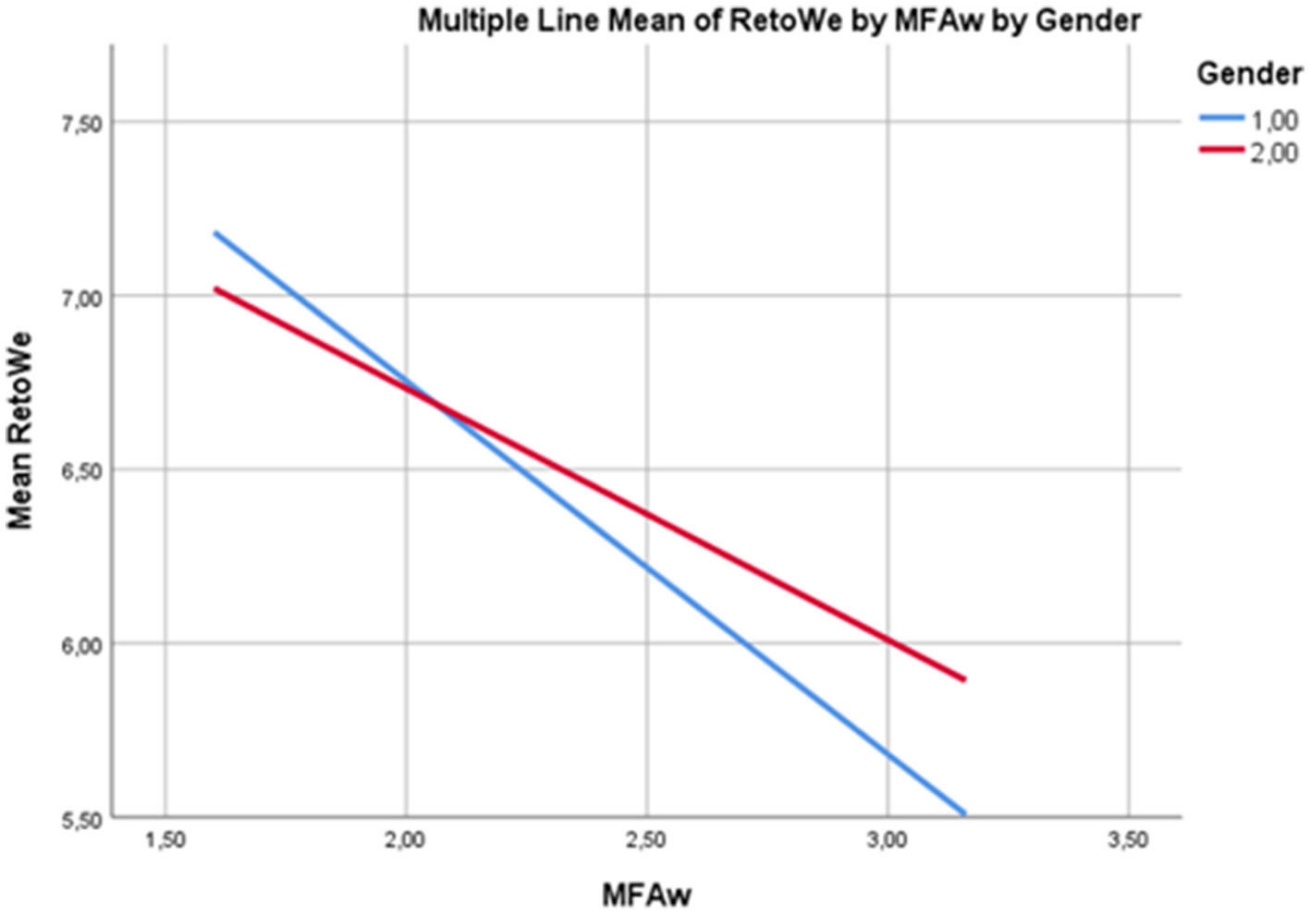
Moderating effect of alcohol consumption in athletes.

Figure 4 illustrates the complex relationships between alcohol consumption, mental fatigue awareness, cognitive reappraisal, and return to well-being in athletes. The analysis revealed a negative association between alcohol consumption and mental fatigue awareness (β = −0.13), which suggests that athletes who consume alcohol may be less aware of their mental fatigue. Interestingly, there was no direct effect of alcohol use on cognitive reappraisal (β = −0.05). However, the study did find several significant indirect relationships. Mental fatigue awareness negatively predicted cognitive reappraisal (β = −0.18), meaning that athletes with higher mental fatigue awareness were less likely to utilize cognitive reappraisal strategies. Conversely, cognitive reappraisal positively predicted return to well-being (β = 0.49), indicating its importance in the recovery process. As previously noted, mental fatigue awareness also had a direct negative effect on return to well-being (β = −0.25). While alcohol consumption appeared to indirectly influence these processes, it did not have a direct effect on return to well-being (β = −0.01). These findings highlight the complex interplay between alcohol consumption, mental fatigue, cognitive strategies, and recovery in athletes.

The analysis results presented in Table 6 evaluate the indirect and total effects of Mental Fatigue Awareness (M_F) and alcohol use (Alcohol1) on Return to Well-being (Re_to_We) through Cognitive Reappraisal (Co_Re).

**Table 6:** Indirect and total effects of mental fatigue awareness and alcohol consumption on well-being through cognitive reappraisal.

**Type**	**Effect**	**Estimate**	**SE**	**Lower**	**Upper**	**β**	***z***	***p***
Indirect	*M_F* → *Co_Re* → *Re_to_We*	−0.244	−0.05	−0.35	−0.13	−0.090	−4.49	<0.001
	*Alcohol1* → *Co_Re* → *Re_to_We*	−0.109	0.08	−0.27	0.06	−0.024	−1.26	0.206
Component	*M_F* → *Co_Re*	−0.317	0.06	−0.44	−0.18	−0.184	−4.71	<0.001
	*Co_Re* → *Re_to_We*	0.770	0.05	0.67	0.87	0.490	15.10	15.10
	*Alcohol1* → *Co_Re*	−0.141	0.11	−0.36	0.07	−0.049	−1.26	0.204
Direct	*M_F* → *Re_to_We*	−0.684	0.08	−0.85	−0.51	−0.253	−7.74	<0.001
	*Alcohol1* → *Re_to_We*	−0.033	0.14	−0.31	0.24	−0.007	−0.23	0.818
Total	*M_F* → *Re_to_We*	−0.928	0.10	−1.12	−0.73	−0.344	−9.17	<0.001
	*Alcohol1* → *Re_to_We*	−0.142	0.16	−0.47	0.18	−0.031	−0.84	0.396

* (M_F), Mental Fatigue Awareness; (Co_Re), Cognitive Reappraisal; (Re_to_We), Return to Well-Being; (Alcohol1), Alcohol Consumption.

#### 3.1.3 Indirect effects

Mental Fatigue Awareness had a significant negative indirect effect on Return to Well-being through Cognitive Reappraisal (Estimate = −0.244, β = −0.090, *p* < 0.001). This indicates that increased awareness of mental fatigue negatively influences the process of returning to well-being via cognitive reappraisal. The indirect effect of alcohol consumption on Return to Well-being through Cognitive Reappraisal was not statistically significant (Estimate = −0.109, β = −0.024, *p* = 0.206), suggesting that alcohol consumption does not have an indirect effect on this process.

#### 3.1.4 Direct effects

Mental Fatigue Awareness had a significant negative direct effect on Return to Well-being (Estimate = −0.684, β = −0.253, *p* < 0.001), demonstrating that increased awareness of mental fatigue directly and negatively affects the return to well-being process. In contrast, the direct effect of alcohol consumption on Return to Well-being was not significant (Estimate = −0.033, β = −0.007, *p* = 0.818). Cognitive Reappraisal showed a positive and strong direct effect on Return to Well-being (Estimate = 0.770, β = 0.490, *p* < 0.001). This finding highlights that the effective use of cognitive reappraisal strategies positively supports the return to well-being process. Additionally, Mental Fatigue Awareness negatively and significantly affected Cognitive Reappraisal (Estimate = −0.317, β = −0.184, *p* < 0.001), indicating that increased mental fatigue awareness negatively influences cognitive reappraisal levels.

#### 3.1.5 Total effects

The total effect of Mental Fatigue Awareness on Return to Well-being was significant and negative (Estimate = −0.928, β = −0.344, *p* < 0.001), showing that mental fatigue awareness negatively impacts the return to well-being process both directly and indirectly. However, the total effect of alcohol use on Return to Well-being was not significant (Estimate = −0.142, β = −0.031, *p* = 0.396), indicating that alcohol use does not have a significant overall impact on this process.

These results clearly demonstrate that mental fatigue awareness negatively affects the return to well-being process, both directly and indirectly through cognitive reappraisal. In contrast, alcohol consumption does not have a significant effect on these processes. The positive effect of cognitive reappraisal on return to well-being emphasizes the supportive role of these strategies in facilitating individuals' return to a state of well-being.

## 4 Discussions

This research looked into the complicated relationship between being aware of mental fatigue and psychological recovery, focusing on the part that cognitive reappraisal plays in controlling emotions. Furthermore, the moderating effects of gender and alcohol consumption were examined to determine their influence on these relationships. This section discusses the results and their implications for theory and practice. The discussion is organized around direct effects, indirect (mediated) effects, and moderation effects.

The results showed that mental fatigue awareness had a significant negative effect on the return to well-being (β = −0.2526, *p* < 0.001, beta = −0.2526, *p* < 0.001, β = −0.2526, *p* < 0.001), which supported Hypothesis 1. A lot of research has shown that mental fatigue lowers motivation, cognitive resources, and emotional resilience, which makes it harder to get better mentally (Pageaux and Lepers, 2018; Russell et al., 2019; Schampheleer and Roelands, 2024). Van Cutsem and Marcora (2021) also emphasize that heightened awareness of mental fatigue exacerbates the perceived burden of fatigue, further impairing overall well-being. These findings suggest that mental fatigue not only influences immediate cognitive performance but also has a prolonged negative impact on an athlete's recovery process, affecting their ability to regain emotional and psychological stability after exertion.

An important finding of the study was that mental fatigue awareness had a negative direct effect on cognitive reappraisal (β = −0.0875, *p* < 0.001, beta = −0.0875, *p* < 0.001, β = −0.0875, *p* < 0.001), which supports Hypothesis 2. This suggests that increased mental fatigue impairs adaptive emotion regulation, making it more challenging for athletes to reinterpret stressors in a constructive manner. This finding is consistent with Lazarus and Folkman's (1986) Stress and Coping Theory, which posits that cognitive appraisal plays a fundamental role in shaping an individual's response to stress (Biggs et al., 2017). It seems that mental fatigue makes it harder for athletes to use cognitive reappraisal effectively, which may make the emotional effects of competitive stressors worse (Nicholls et al., 2012). More proof that cognitive reappraisal has a positive and significant direct effect on return to well-being (β = 0.4909, *p* < 0.001, beta = 0.4909, *p* < 0.001, β = 0.4909, *p* < 0.001), which supports Hypothesis 3. This supports the idea that cognitive reappraisal is an important psychological process that helps people recover from stress caused by fatigue. It also backs up earlier research that found emotion regulation strategies to be important for athletes' psychological resilience (van Rens et al., 2021; Riepenhausen et al., 2022).

The study of indirect effects showed that cognitive reappraisal played a part in the link between mental fatigue awareness and return to well-being (β = −0.236, *p* < 0.001, beta = −0.236, *p* < 0.001). This means that cognitive reappraisal may help lessen the negative effects of mental fatigue, but it may not fully explain the relationship. This means that other things may also play a role in the recovery process. The full study also showed that 74.3% of the effect of mental fatigue awareness on recovery was direct, while only 25.7% was mediated by cognitive reappraisal. These results show that even though strategies for controlling emotions are important for mental health recovery, mental fatigue is still the main issue that needs more ways to deal with than just cognitive reappraisal.

One intriguing thing that this study showed was that gender played a moderating role in the relationship between mental fatigue awareness and recovery. It showed that this effect was stronger in female athletes, who had a worse time recovering than male athletes. This finding fits with the Emotional Processing Theory (Foa et al., 2006), which says that people have different ways of handling and controlling their emotions, which can change how they act when they are stressed (Baker et al., 2007). Research has shown that women tend to process emotional information in greater depth than men (Brody and Hall, 2008; Fiorentini, 2013; Chaplin, 2015). Consequently, heightened awareness of mental fatigue may intensify perceived stress in female athletes, making it more challenging for them to disengage from fatigue-related distress. In contrast, male athletes, who may adopt a more pragmatic or less cognitively elaborate approach to emotional processing, appear to be less affected by mental fatigue awareness in their recovery processes. The discovery of gender as a moderator in the relationship between mental fatigue awareness and recovery is a novel development in the field of sport psychology. As far as we know, no other study has specifically looked into this relationship. This finding underscores the importance of recognizing gender as not merely a biological characteristic but as a psychological factor that shapes emotional processing and resilience in athletic contexts. The results suggest that gender differences in mindfulness, emotion regulation, and attentional disengagement from stressors may play a pivotal role in recovery processes among athletes.

Contrary to expectations, the study did not reveal a statistically significant moderating effect of alcohol consumption on the relationship between mental fatigue awareness and recovery. Specifically, alcohol use showed no significant direct or indirect effect on return to well-being through cognitive reappraisal. This finding suggests that, within the scope of this study, alcohol consumption does not exert a substantial influence on athletes' recovery processes. It fits with the Cognitive Appraisal Theory (Smith and Kirby, 2012), which says that how someone subjectively judges stressors determines their emotional and mental responses, regardless of outside factors like drinking alcohol (Moors, 2020). In general, drinking alcohol has been linked to problems with controlling emotions and executive functioning (Zhou et al., 2016; Knettel et al., 2021). However, this study suggests that these problems may not have a big effect on cognitive reappraisal or psychological recovery in athletes. The unexpected nature of this finding raises important questions for future research. It is possible that alcohol's impact on recovery is dependent on additional moderating factors such as consumption patterns, individual tolerance levels, or contextual stressors associated with athletic performance. Further studies should explore how different levels of alcohol consumption (e.g., occasional vs. chronic use) influence cognitive and emotional recovery mechanisms in sports contexts.

This study adds to what's already been written by looking at how gender and alcohol use affect the mental fatigue-recovery process. Sport psychology hasn't extensively explored these areas. While prior research has emphasized the detrimental effects of alcohol on cognitive and emotional regulation, the present study suggests that its influence on recovery processes in athletes may not be as straightforward as previously assumed. This novel finding highlights the need for more granular investigations into how specific drinking behaviors, personality traits, and situational stressors interact with mental fatigue and emotion regulation strategies.

This study shows how important cognitive reappraisal is as a psychological process that helps people recover from mental fatigue. It also shows how gender affects how people process their emotions in sports situations. The results suggest that interventions that want to speed up the recovery process should include specific plans that take into account differences between people in how they handle their emotions, how they process information based on their gender, and their overall psychological resilience. While alcohol consumption did not emerge as a significant moderator, future research should investigate how different drinking patterns and situational contexts influence psychological recovery in competitive sports. By deepening our understanding of these complex relationships, this study provides a valuable foundation for future research in mental fatigue, emotion regulation, and resilience in athletic performance.

## 5 Conclusions

This study examined the relationship between mental fatigue awareness and return to well-being, emphasizing the mediating role of cognitive reappraisal. The results show that being more aware of mental fatigue makes it much harder to get back to a state of well-being. Cognitive reappraisal helps to moderate this relationship, but it doesn't fully reverse the negative effects.

A key finding was the moderating role of gender, with female athletes experiencing greater difficulties in returning to well-being compared to male athletes. This highlights the need for gender-sensitive psychological interventions. Contrary to expectations, alcohol consumption did not significantly moderate the relationship between mental fatigue awareness and return to well-being, suggesting that its influence may be contingent on other contextual or individual factors.

The results align with Stress and Coping Theory, reinforcing the importance of cognitive reappraisal in return to well-being. As a result, targeted interventions are being created to improve cognitive reappraisal skills, especially for people who are more aware of their mental fatigue. Gender-specific approaches should be integrated into psychological support programs to address differences in emotional processing.

Future research should adopt longitudinal designs to establish causal relationships and examine additional moderating factors such as sleep quality, nutrition, and training load. By focusing on the specific subdimensions of emotion regulation and psychological recovery, this study contributes to a more nuanced understanding of mental fatigue awareness and psychological recovery in competitive sports.

## 6 Limitations

While this study provides valuable insights into the relationships between mental fatigue, cognitive reappraisal, and recovery processes in athletes, it is important to acknowledge its limitations.

- *Sampling Method*: The convenience sampling method employed limits the generalizability of the findings. Although athletes from various regions of Turkey participated, the sample lacked demographic and sports homogeneity. Future research with a more diverse and representative sample is warranted.
- *Cross-Sectional Design*: The cross-sectional nature of the study precludes the establishment of causal relationships between variables. Longitudinal studies are needed to examine the temporal dynamics and causal pathways involved in mental fatigue, cognitive reappraisal, and recovery.
- *Alcohol Consumption and Gender Variables*: The assessment of alcohol use relied on limited measures, restricting the ability to capture detailed information regarding consumption levels, types, and patterns. Besides, the study did not fully explore the social and cultural dimensions of gender.
- *Self-Report Scales*: Reliance on self-report measures introduces the potential for subjective biases, such as social desirability bias, which may affect the accuracy of the data.
- *Online Data Collection*: The online data collection method limited the ability to observe athletes' physical and mental states directly and restricted data validation processes.

## 7 Recommendations

To address these limitations and improve this line of inquiry, the following recommendations are proposed:

*Longitudinal Research*: Future studies should employ longitudinal designs to investigate the long-term effects and causal relationships between mental fatigue awareness, cognitive reappraisal, and recovery processes.

*Diversified Sampling*: Research should aim for greater sample diversity, including athletes of different ages, competitive levels, and sports disciplines, as well as athletes from different countries and cultural backgrounds.

*Qualitative Data*: Incorporating qualitative methods, such as in-depth interviews and focus groups, can provide rich insights into the lived experiences of athletes and enhance understanding of mental fatigue, recovery, and cognitive reappraisal processes.

*Detailed Investigation of Alcohol Consumption*: Future research should utilize more comprehensive measures of alcohol use, capturing details about consumption levels, patterns, and types. Experimental studies could also be conducted to examine the specific psychological and physiological effects of alcohol consumption on athletes.

*Inclusion of Contextual Factors*: A more holistic approach should be adopted, considering not only the biological but also the social, cultural, and environmental dimensions of gender and their potential influence on the studied processes. Factors such as training intensity, competition level, and social support mechanisms should also be investigated.

*Guidelines for Practical Applications*: The findings of this research can inform the development of training programs and guidelines for sport psychology consultants, coaches, and athletes to enhance mental fatigue awareness, optimize recovery processes, and cultivate effective cognitive reappraisal skills.

*Age as a Confounding Variable*: Future research should examine the moderating effect of age on mental fatigue awareness. Age may be a determinant variable in terms of cognitive processes and stress management. This effect was not tested in the current study, but more comprehensive results can be obtained by analyzing the indirect and direct effects of age with advanced statistical methods such as structural equation modeling (SEM) in future studies. In particular, comparing different age groups will contribute to the development of customized strategies to increase awareness of mental fatigue.

## Data Availability

The original contributions presented in the study are included in the article/supplementary material, further inquiries can be directed to the corresponding author.
